# Incidence and predictors of tuberculosis among HIV-positive children at Adama Referral Hospital and Medical College, Oromia, Ethiopia: a retrospective follow-up study

**DOI:** 10.4178/epih.e2019028

**Published:** 2019-06-22

**Authors:** Masino Tessu Beshir, Aklil Hailu Beyene, Kenean Getaneh Tlaye, Tefera Mulugeta Demelew

**Affiliations:** 1 Department of Nursing, Wolkite University College of Health Science and Medicine, Wolkite, Ethiopia; 2 Department of Nursing, Addis Ababa University College of Health Science, Addis Ababa, Ethiopia; 3 Department of Nursing, Woldia University Faculty of Health Science, Woldia, Ethiopia

**Keywords:** Incidence, Tuberculosis, Children, HIV, Ethiopia

## Abstract

**OBJECTIVES:**

Tuberculosis (TB) is common in children with human immunodeficiency virus (HIV), but its effect on the survival of HIV-infected children is not well understood. Therefore, the aim of this study was to assess the incidence and predictors of active TB among HIV-positive children at Adama Referral Hospital and Medical College, Oromia, Ethiopia.

**METHODS:**

A retrospective study was conducted over 5 years using a checklist to gather data from 428 randomly selected pediatric patient charts. The checklist was adapted from the standardized antiretroviral therapy (ART) follow-up form currently used by the institution’s ART clinic. Data were analyzed by bivariate and multivariable analysis using Cox regression proportional hazards models, as appropriate. Survival was calculated and compared using the Kaplan-Meier and log-rank tests.

**RESULTS:**

Of the 466 charts reviewed, 428 patient records were included in the analysis. A total of 67 new TB cases were observed during the follow-up period. Hence, the incidence rate in this cohort was found to be 6.03 per 100 child-years of observation. A baseline hemoglobin level <10 g/dL (adjusted hazard ratio [aHR], 7.04; 95% confidence interval [CI], 1.03 to 48.15), moderate wasting (aHR, 2.86; 95% CI, 1.02 to 7.99), and not receiving isoniazid preventive therapy (aHR, 8.23; 95% CI, 2.11 to 32.06) were among the independent predictors of TB occurrence.

**CONCLUSIONS:**

The incidence of TB was high, particularly in pre-ART patients receiving chronic care for HIV. Close followup of HIV-positive children is crucial to protect them against the development of TB. Initiating isoniazid preventive therapy, averting malnutrition, and managing anemia are also of significant importance.

## INTRODUCTION

Tuberculosis (TB) is a leading cause of death and a common illness among people living with human immunodeficiency virus (HIV). At least 1 in 4 HIV-related deaths is a result of TB, and many of these deaths occur in developing countries [1]. People living with HIV are around 30 times more likely to develop TB than HIV-negative people. HIV impairs T-cell–related immunity, which predisposes those with HIV infection to TB infection [1,2]. There were an estimated 1 million new cases of TB in children aged 0-14 in 2014, accounting for approximately 10% of the total TB burden [3].

The 2015 worldwide report on the global burden of TB mortality in children showed that TB was responsible for more deaths than other infectious diseases, such as HIV. In 2015 an estimated 1.4 million HIV-negative and 400,000 HIV-positive individuals died of TB. Among those, 239,000 were children [4]. Childhood TB is often called “the hidden epidemic” due to the difficulties involved in finding and treating the disease in this population, in both developed and developing countries. In settings with a high burden of TB, children account for an estimated 15-20% of TB cases [5,6].

TB is largely a disease of poverty, and children with the disease frequently live in poor communities with few health services [7]. In African sub-regions, various risk factors for mortality have been identified among children with TB-HIV coinfection, before or during antiretroviral therapy (ART); some of these children had received anti-TB treatment prior to ART initiation, while others had not [8]. Ethiopia ranks seventh among the world’s 22 high-TB-burden countries in which prevention and control pose additional challenges to the health care system [9]. Moreover, resources for pediatric TB diagnosis and treatment are usually restricted to higher-level care facilities, such as national referral hospitals and facilities in urban settings [10,11].

TB transmission in health care settings, at home, and in the community is one of the primary research questions in the area of TB prevention. To improve child survival before and after the initiation of ART, a better understanding of risk factors and disease epidemiology in this pediatric population is crucial. Therefore, the major aim of this study was to assess the incidence and predictors of TB among children living with HIV and receiving follow-up care at Adama Referral Hospital and Medical College, Oromia, Ethiopia.

## MATERIALS AND METHODS

### Study design, setting, and period

A 5-year institution-based retrospective study was conducted. The study was conducted at the HIV/AIDS care clinic of Adama Referral Hospital and Medical College from February 28, 2018 to March 13, 2018. The town of Adama, located 99 km southeast of Addis Ababa, is a special administrative Zone of Oromia Regional State. Adama Hospital and Medical College was one of the first 3 hospitals in Oromia to provide ART services, starting in 2005. Routine care for HIV-positive children at the ART clinic includes screening for opportunistic infections, evaluation of clinical staging, and assessment of eligibility for ART by trained clinicians. Patients are managed in accordance with World Health Organization (WHO) recommendations [12]. The ART case team in the hospital is composed of ART-trained physicians, ART-trained nurses, pharmacists, laboratory technicians, data clerks, and drug adherence counselors. A total of 1,309 children have been enrolled since March 2005 and 633 children have started ART since January 2013 [13]. The study subjects were followed from January 1, 2013 to December 31, 2017.

### Population and sampling

The study population consisted of all HIV-positive children less than 14 years of age registered with the chronic HIV care and support program at Adama Referral Hospital from January 1, 2013 to December 31, 2017. This study period was selected because the facility started full implementation of a standardized formatting, documentation, and recording system in a regular manner in 2013. The inclusion criteria included being a newly enrolled patient during the study period. Subjects received TB screening through a combination of an evaluation of signs and symptoms, laboratory tests, and an X-ray examination, and were confirmed to be TB-free. Patients with incomplete baseline information such as CD4 count and hemoglobin levels, as well as HIV-positive children who had already started anti-TB treatment at the beginning of the follow-up, were excluded.

### Sample size determination

As the study was designed to determine the incidence of TB and its predictors, the authors considered both objectives to calculate the largest sample size needed. For the first objective, a single-population proportion formula was used to calculate the sample size by considering the proportion of TB incidence among HIV-positive children (20%) [14], which yielded 270. For the second objective, the sample size was determined using a double-population proportion formula by considering anemia, ambulatory functional status, and not having been vaccinated as major predictor variables. Among these variables, ambulatory functional status generated the maximum sample size, with 25.0% of exposed individuals having the outcome and 13.8% of non-exposed individuals having the outcome [14]. Therefore, the largest calculated sample size (n=466) was selected as the final sample size for the study.

### Sampling procedures and study variables

To select the study cohort, a simple random sampling technique was applied to the list of 633 HIV-infected children who had started ART at the ART unit. The primary outcome was TB incidence and time to occurrence. Other variables of interest extracted from chart review included: age, sex, residence, parental status, family size, caregiver, WHO clinical HIV stage, CD4 count, hemoglobin level, functional status, initial ART regimen, prior history of TB treatment, isoniazid preventive therapy (IPT), cotrimoxazole preventive therapy (CPT), Bacillus Calmette–Guérin (BCG) vaccination status, and nutritional status.

### Operational definitions

#### Entry date

The first date for each observation within the study period at the date of HIV/AIDS confirmation.

#### End date

The last date for each observation within the study period, corresponding to the subject’s last visit.

#### Survival

The time from date of HIV/AIDS confirmation to the occurrence of TB.

#### Events

Incident TB, which was defined in this study as the occurrence of active TB in children living with HIV any time after the children were enrolled into chronic HIV care. The type of TB could be smear-positive pulmonary, smear-negative pulmonary, or extrapulmonary TB as identified by signs and symptoms, laboratory tests, X-ray examinations, and/or the initiation of anti-TB treatment.

#### Censored

Children living with HIV were censored at the first date of loss to follow-up, drop-out, transferring out, or death by other causes before the end of the follow-up period. Additionally, those who completed the follow-up period without developing the event were censored.

#### Stunting, underweight, and wasting

The child being 2 standard deviations (SDs) below the norm for height for age, weight for age, or weight for height, according to the WHO 2006 curve.

#### Bacillus Calmette–Guérin vaccination status

Subjects were categorized as vaccinated or not vaccinated by inspecting them for the presence of a BCG scar.

### Data collection process and procedures

The data extraction tool was developed based on the format of the WHO standardized ART entry and follow-up form currently used by the ART clinic. Data were collected via review of patients’ charts and ART cards. Deaths were confirmed by reviewing medical registration in the hospital, or through registration by an ART adherence supporter via calling the registered phone number. The most recent laboratory test results before starting ART were used as a baseline value. If there were no registered pre-ART laboratory tests, results obtained within 1 month of ART initiation were used as a baseline. If 2 results were obtained within 1 month, the mean value was used.

### Data quality control

Three nurses (bachelor degree holders) from Adama Referral Hospital and Medical College, who were trained in comprehensive HIV care and currently involved in patient follow-up care, were employed as data collectors. Both the principal investigator and their supervisors in the hospital closely supervised the data collection process. A 1-day training session was given concerning the data extraction tool and the data collection process for both data collectors and supervisors.

### Data processing and analysis

Data was entered using Epidata version 4.2.0.0 (http://www.epidata.dk/index.htm) and the analysis was done using Stata version 14.1 (StataCorp., College Station, TX, USA). The ENA for SMART 2011 (http://www.nutrisurvey.de/ena2011/) was used to generate Z-scores to define participants’ nutritional status. Before analysis, the data were cleaned and checked for completeness. The assumptions of Cox proportional hazards regression models were checked using the Schoenfeld test and the residual test, and variables with a p-value >0.1 were considered to have fulfilled the assumptions. The outcome of each participant was dichotomized as censored or incidence of TB. Kaplan-Meier survival curves were used to estimate the time of TB occurrence among HIV-positive children, and the log-rank test was used to compare survival curves. A bivariate Cox proportional hazards regression model was fitted for each explanatory variable. Moreover, variables with a p-value ≤0.25 in the bivariate analysis were fitted to the multivariable Cox proportional hazards regression model. Hazard ratios (HRs) with 95% confidence intervals (CIs) and p-values were used to measure the strength of associations and to identify statistically significant results. In the multivariable analysis, variables with a p-value <0.05 were considered to be significant predictors of TB incidence after enrollment in HIV chronic care.

### Ethics statement

Ethical clearance was obtained from Addis Ababa University School of Nursing and Midwifery research committee. Permission letter was obtained from hospital administration. As this is a retrospective study, informed consent from individual patients was not requested. Since, the study was done through reviewing of medical records, the individual patients might not be subjected to harm as much as the confidentiality was kept. To keep the confidentiality all collected data was coded and locked in a separate room before entered into the computer. After entered to the computer the data was locked by password, names and unique ART numbers was not included in the data collection format and the data was not disclosed to any person other than principal investigator.

## RESULTS

### Socio-demographic characteristics of the study participants

Among the 466 records of HIV-positive children that were reviewed, 428 (91.8%) records were included in the final analysis. Thirty-eight (8.2%) children were excluded from the analysis because of incomplete documentation. The median age of the cohort at the time of HIV diagnosis was 6 years, with an interquartile range (IQR) of 3-10 years. Roughly half (51.9%) of the study participants were males, and 245 (57.2%) of them were above the age of 5 (Table 1).

### Baseline clinical, laboratory, and antiretroviral therapy information

The eligibility criteria for initiation of ART were mainly both CD4 count and WHO clinical stage. More than half (55.4%) of the participants were at WHO clinical stage I or II, and 124 (29.0%) had a CD4 count below the threshold for severe immunodeficiency at ART initiation. Nearly half of the children had an ambulatory functional status at baseline (49.6%) and more than half of the children had a delayed developmental history (53.2%). Nearly three-fourths of the children (n=313, 73.1%) had hemoglobin levels less than 10 g/dL. During the follow-up period, most of children (93.6%) received CPT, but only 154 (42.4%) received IPT. Fifty-five (82.1%) TB patients presented with pulmonary TB, followed by 12 (17.9%) with extrapulmonary TB, making the incidence rate of pulmonary and extrapulmonary TB 4.5 and 1 per 100 child-years of observation, respectively. Forty-eight patients (71.6%) developed TB for the first time during the pre-ART period, while 19 (28.4%) developed TB during ART (Table 2).

Regarding the ART regimen given for HIV-positive children, the predominant regimen initially prescribed was a combination of zidovudine, lamivudine, and nevirapine (4c=AZT-3TC- NVP) in 195 patients (45.6%), followed by zidovudine, lamivudine, and efavirenz (4d=AZT-3TC-EFV) in 152 patients (35.5%).

### Nutritional characteristics

When their nutritional status was analyzed relative to their age, approximately a quarter of the HIV-positive children (26.2%) were underweight, 73 (17.0%) showed wasting, and 67 (15.6%) were stunted (Table 3).

### Tuberculosis incidence after human immunodeficiency virus confirmation

**Figure 1.**
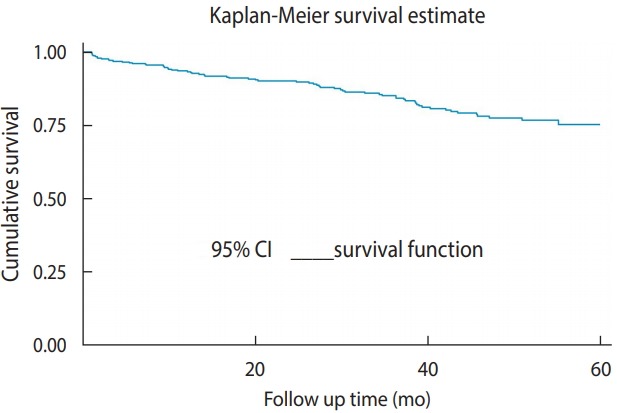
Overall Kaplan-Meier survival curve with 95% confidence intervals (CIs) for tuberculosis-free survival among children receiving chronic human immunodeficiency virus care at Adama Referral Hospital and Medical College.

A total of 428 children included in the study were followed for up to 60 months. The median follow-up duration was 32.5 months (IQR, 12.30 to 50.13), and the minimum and the maximum follow-up times were 0.63 and 60 months, respectively. Of the total observations, 275 (64.2%) were alive at the end of follow-up, 69 (16.1%) had died, 60 (14.0%), had been lost to follow-up, and 24 (5.6%) had been transferred out. The incidence rate in this cohort was found to be 6.03 per 100 child-years of observation. There were 67 new TB cases observed during follow-up. The incidence rate of TB during the pre-ART and ART periods was 5.97 and 2.36 per 100 person-years observation, respectively. The cumulative survival probabilities at 6, 12, 18, 36, and 60 months after confirmation of HIV status were 0.96, 0.93, 0.91, 0.84, and 0.75, respectively. The mean survival time of the entire cohort was found to be 31.10 months (95% CI, 29.20 to 33.01) and the cohort experienced a total of 13,314.63 person-months of follow-up (Figure 1).

### Survival function according to cotrimoxazole preventive therapy

To test the equality of the survival curves obtained using different categorical explanatory variables, the Cochran-Mantel-Haenszel log-rank test was performed. The test statistics showed that there was a significant difference in survival function for different categorical variables. One of these differences was observed among those who received CPT and those who did not.

In this historical cohort, the study participants who did not receive CPT had a shorter survival time than their counterparts (p< 0.001).

**Figure 2.**
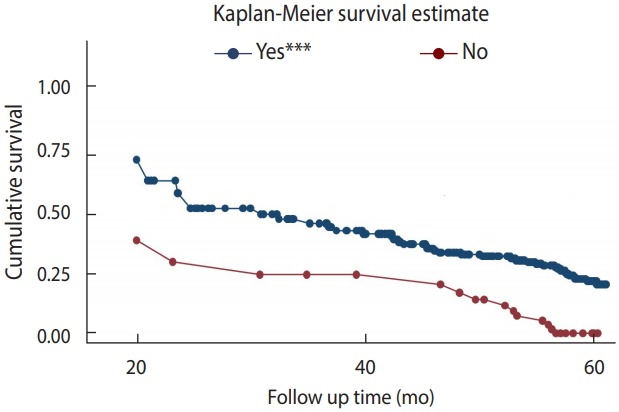
Kaplan-Meier survival curve for tuberculosis-free survival based on cotrimoxazole preventive therapy among children receiving chronic human immunodeficiency virus care at Adama Referral Hospital and Medical College (n=362). ***p<0.001.

The mean±SD survival time for those who received CPT was found to be 55.10±0.90 months, as compared to 34.91±4.18 months in those who did not receive CPT. This difference was statistically significant (p<0.001) (Figure 2).

### Predictors of tuberculosis occurrence

The relationships between the baseline variables and the incidence of TB were analyzed using a bivariate Cox proportional hazards regression model. The results of the bivariate analysis showed associations with TB incidence for a wide range of factors, including an age of 6-10 years, living in an urban setting, a family size ≥3, hemoglobin levels <10 g/dL, ambulatory and bedridden functional status, WHO clinical stages III and IV, underweight (moderate and severe), stunting (moderate), wasting (moderate and severe), not receiving CPT or IPT, and not being vaccinated. However, sex, CD4 count, and the remaining 3 variables (parental status, care givers, and developmental history) did not show significant associations. Considering the threshold of a p-value ≤0.25, these variables were not entered into the multivariable Cox proportional hazards model.

In the multivariable Cox proportional hazards model, only 6 variables were associated with the incidence of TB. The multivariable analysis revealed that children who had not taken cotrimoxazole at baseline were at a 2.41 times higher risk of developing TB than those who had taken cotrimoxazole at baseline (adjusted hazard ratio [aHR], 2.41; 95% CI, 1.07 to 5.45). Children who did not receive IPT were at an 8.23 times higher risk of developing TB than those who did (aHR, 8.23; 95% CI, 2.11 to 32.06). The risk of developing TB in children who had not received the BCG vaccine was 3.73 times higher than in their counterparts who had been vaccinated (aHR, 3.73; 95% CI, 1.59 to 8.76).

Children with a hemoglobin level less than 10 g/dL were at a 7.04 times higher risk of developing TB than those with a hemoglobin level greater than or equal to 10 g/dL (aHR, 7.04; 95% CI, 1.03 to 48.15). Children who were moderately underweight at the beginning of ART were at a 5.19 higher risk of developing TB than those who were not underweight (aHR, 5.19; 95% CI, 1.89 to 14.21). Furthermore, children with moderate wasting were at a 2.86 times higher risk of developing TB than those with no wasting (aHR, 2.86; 95% CI, 1.02 to 7.99) (Table 4).

## DISCUSSION

The TB incidence rate observed in this study (6.03 per 100 child-years) is consistent with 2 studies conducted in northern Ethiopia and 1 conducted in Tanzania, which reported rates of 4.2, 4.9, and 5.2 per 100 child-years respectively [14-16]. In our study, HIV-positive children with baseline anemia had a 7.04 times higher risk of developing TB than non-anemic children. This finding is similar to those of studies conducted in northern Ethiopia [14,15] and Tanzania [16].

Our study showed that children who did not receive IPT were at a 8.23 times higher risk of developing TB than their counterparts. This finding is also similar to that of a previous study done in northern Ethiopia [15]. This could be due to the fact that IPT decreases the mycobacterium load and reduces the progression of latent bacilli to active TB [17].

BCG vaccination status was also found to be a significant predictor in our study, as children who had not been vaccinated had a nearly 4-fold higher risk of TB than their vaccinated counterparts. This finding is similar to the results of a study from Tanzania. It is also supported by prior studies showing that BCG vaccination considerably reduced the risk of TB among individuals with and without HIV infection [14,18]. Underweight was also found to be an independent predictor of TB incidence. This finding is consistent with those of studies done in Kenya and the Democratic Republic of Congo [19,20].

Limitations of this study include the fact that some data, such as history of TB exposure, were frequently missing and therefore could not be included in the analysis. It was also not possible to measure some data such as household income, housing condition, HIV viral load, and other potentially important predictors of TB.

In conclusion, there was a high rate of early incidence of TB among children living with HIV, especially in their first year of enrollment in chronic HIV care. The incidence of TB was high before ART. Baseline malnutrition in the form of moderate underweight and moderate wasting, not receiving CPT or IPT, not being vaccinated, and anemia were all found to be independent predictors of TB occurrence among HIV-positive children. This might have been due to the fact that 81.1% of our study participants took ART regimens of 4c (AZT-3TC-NVP) or 4d (AZT-3TC-EVF) during their follow-up period. AZT is known to be one of the most common causes of megaloblastic anemia, which is in turn closely associated with pancytopenia. This condition imposes a double burden on children with HIV and makes them vulnerable to infections, including TB.

We recommend that TB/HIV collaborative long-term surveillance programs should be strengthened at chronic HIV care clinics. Further prospective studies should also be conducted by incorporating important predictors of TB occurrence, such as viral load.

## Figures and Tables

**Table 1.** Baseline socio-demographic characteristics of children living with HIV treated at Adama Referral Hospital and Medical College

Variables	n (%)
Sex	
Male	222 (51.9)
Female	206 (48.1)
Age (yr)	
≤5	183 (42.8)
6-10	153 (35.7)
≥11	92 (21.5)
Residence	
Urban	293 (68.5)
Rural	135 (31.5)
Family size (n)	
≤2	84 (19.6)
3-4	242 (56.6)
≥5	102 (23.8)
Caregiver of the child	
Parents	375 (87.6)
Sibling	12 (2.8)
Grandparent	26 (6.1)
Guardians	13 (3.0)
Orphanage centers	2 (0.5)

HIV, human immunodeficiency virus.

**Table 2.** Baseline clinical, laboratory, and ART information for children living with HIV/AIDS treated at Adama Referral Hospital and Medical College

Variables	n (%)
Functional status (age ≥5 yr) (n=270)	
Working	123 (45.6)
Ambulatory	134 (49.6)
Bedridden	13 (4.8)
Developmental history (age <5 yr) (n=158)	
Appropriate	65 (41.1)
Delayed	84 (53.2)
Regressive	9 (5.7)
WHO clinical stage	
I or II	237 (55.4)
III or IV	191 (44.6)
CD4 count or percent	
Below the threshold	124 (29.0)
Above the threshold	304 (71.0)
Hemoglobin level (g/dL)	
<10	298 (69.6)
≥10	130 (30.4)
ART eligibility criteria	
CD4 cell count	121 (28.3)
WHO clinical stage	50 (11.7)
Both	171 (39.9)
Not recorded	86 (20.1)
Cotrimoxazole preventive therapy (n=362)	
Yes	339 (93.6)
No	23 (6.3)
Isoniazid preventive therapy (n=362)	
Yes	154 (42.5)
No	208 (57.5)
Types of TB (n=67)	
Pulmonary TB	55 (82.1)
Extrapulmonary TB	12 (17.9)
Time TB developed (n=67)	
Pre-ART	48 (71.6)
ART	19 (28.4)

ART, antiretroviral therapy; WHO, World Health Organization; TB, tuberculosis.

**Table 3.** Baseline nutritional status of HIV-positive children categorized by age at Adama Referral Hospital and Medical College

Nutritional parameter	Age (yr)			Total (n=428)
	≤5 (n=183)	6-10 (n=153)	≥11 (n=92)	
Normal WFA	144	125	47	316
Moderate underweight (< -2 SD)	30	17	39	86
Severe underweight (< -3 SD)	9	11	6	26
Normal HFA	162	131	68	361
Moderate stunting (< -2 SD)	13	13	20	46
Severe stunting (< -3 SD)	8	9	4	21
Normal WFH	146	119	90	355
Moderate wasting (< -2 SD)	23	28	2	53
Severe wasting (< -3 SD)	14	6	0	20

HIV, human immunodeficiency virus; WFA, weight for age; HFA, height for age; WFH, weight for height; SD, standard deviation.

**Table 4.** Bivariate and multivariable Cox regression proportional hazards regression analysis of predictors of tuberculosis incidence among HIV-infected children at Adama Referral Hospital and Medical College

Variables	Survival status, n (%)		cHR (95% CI)	aHR (95% CI)	p-value
	Censored	Event			
Age (yr)					
≤5	171 (39.9)	12 (2.8)	0.12 (0.06, 0.24)	0.78 (0.92, 1.44)	0.61
6-10	129 (30.1)	24 (5.6)	0.29 (0.16, 0.50)	1.53 (0.80, 1.72)	0.57
≥11	61 (14.2)	31 (7.2)	1.00 (reference)	1.00 (reference)	
Residence					
Urban	237 (55.4)	56 (13.1)	1.00 (reference)	1.00 (reference)	
Rural	124 (29.0)	11 (2.6)	0.40 (0.21, 0.76)	0.84 (0.27, 2.55)	0.78
Family size (n)					
≤2	77 (18.0)	7 (1.7)	1.00 (reference)	1.00 (reference)	
3-4	202 (47.2)	40 (9.3)	2.41 (1.07, 5.39)	2.74 (0.72, 10.44)	0.14
≥5	82 (19.1)	20 (4.7)	2.37 (1.00, 5.62)	2.36 (0.54, 10.15)	0.25
Functional status					
Working	113 (26.4)	10 (2.3)	1.00 (reference)	1.00 (reference)	
Ambulatory	91 (5.6)	43 (10.0)	3.35 (1.68, 6.67)	1.02 (0.39, 2.68)	0.95
Bedridden	8 (1.9)	5 (1.2)	5.82 (1.98, 17.05)	3.01 (0.51, 17.71)	0.22
WHO clinical staging					
I or II	202 (47.2)	35 (8.2)	1.00 (reference)	1.00 (reference)	
III or IV	159 (37.1)	32 (7.5)	1.35 (0.83, 2.19)	1.30 (0.67, 2.51)	0.43
Hemoglobin level (g/dL)					
<10	239 (60.0)	59 (13.1)	3.48 (1.66, 7.29)	7.04 (1.03, 48.15)	0.04
≥10	122 (24.3)	8 (2.6)	1.00 (reference)	1.00 (reference)	
Cotrimoxazole preventive therapy					
Yes	303 (70.8)	36 (8.4)	1.00 (reference)	1.00 (reference)	
No	10 (2.3)	13 (3.0)	6.00 (3.17, 11.36)	2.41 (1.07, 5.45)	0.03
Isoniazid preventive therapy					
Yes	149 (34.8)	5 (1.2)	1.00 (reference)	1.00 (reference)	
No	164 (38.3)	44 (10.3)	8.48 (3.36, 21.44)	8.23 (2.11, 32.06)	0.002
BCG vaccination status					
Vaccinated	211 (49.3)	15 (3.5)	1.00 (reference)	1.00 (reference)	
Not vaccinated	86 (20.1)	48 (11.2)	6.70 (3.74, 11.98)	3.73 (1.59, 8.76)	0.002
Underweight (WFA)					
Normal	290 (67.7)	26 (6.1)	1.00 (reference)	1.00 (reference)	
Moderate underweight	49 (11.4)	37 (8.6)	7.50 (4.52, 12.45)	5.19 (1.89, 14.21)	0.001
Severe underweight	22 (5.1)	4 (0.9)	3.52 (1.22, 10.12)	1.34 (0.00, ...)^1^	1.00
Wasting (WFH)					
Normal	303 (70.8)	52 (12.1)	1.00 (reference)	1.00 (reference)	
Moderate wasting	39 (6.4)	14 (3.3)	2.32 (1.23, 4.04)	2.86 (1.02, 7.99)	0.04
Severe wasting	19 (4.4)	1 (0.2)	0.31 (0.04, 2.29)	2.39 (0.22, 25.19)	0.47

HIV, human immunodeficiency virus; cHR, crude hazard ratio; aHR, adjusted hazard ratio; CI, confidence interval; WHO, World Health Organization; BCG, Bacillus Calmette–Guérin; WFA, weight for age; WFH, weight for height.

1 The lower boundary is zero and the upper is not given by analysis.
